# Measurement and Finite Element Model Validation of Immature Porcine Brain–Skull Displacement during Rapid Sagittal Head Rotations

**DOI:** 10.3389/fbioe.2018.00016

**Published:** 2018-02-21

**Authors:** Stephanie A. Pasquesi, Susan S. Margulies

**Affiliations:** ^1^Injury Biomechanics Laboratory, Department of Bioengineering, University of Pennsylvania, Philadelphia, PA, United States

**Keywords:** finite element model, brain–skull displacement, boundary condition, traumatic brain injury, bridging vein

## Abstract

Computational models are valuable tools for studying tissue-level mechanisms of traumatic brain injury, but to produce more accurate estimates of tissue deformation, these models must be validated against experimental data. In this study, we present *in situ* measurements of brain–skull displacement in the neonatal piglet head (*n* = 3) at the sagittal midline during six rapid non-impact rotations (two rotations per specimen) with peak angular velocities averaging 51.7 ± 1.4 rad/s. Marks on the sagittally cut brain and skull/rigid potting surfaces were tracked, and peak values of relative brain–skull displacement were extracted and found to be significantly less than values extracted from a previous axial plane model. In a finite element model of the sagittally transected neonatal porcine head, the brain–skull boundary condition was matched to the measured physical experiment data. Despite smaller sagittal plane displacements at the brain–skull boundary, the corresponding finite element boundary condition optimized for sagittal plane rotations is far less stiff than its axial counterpart, likely due to the prominent role of the boundary geometry in restricting interface movement. Finally, bridging veins were included in the finite element model. Varying the bridging vein mechanical behavior over a previously reported range had no influence on the brain–skull boundary displacements. This direction-specific sagittal plane boundary condition can be employed in finite element models of rapid sagittal head rotations.

## Introduction

Finite element modeling is a popular computational tool used by many researchers to aid in understanding and prediction of traumatic brain injury (TBI). To simulate injurious occurrences accurately, finite element models must include appropriate representations of the brain, skull, and other cranial contents in terms of geometry, material properties, and contact interactions between different structures. Extra-axial hemorrhage is thought to stem from the rupture or tearing of the parasagittal bridging veins, as the cortex and skull move relative to each other. Thus, the boundary condition between the brain and skull is of utmost importance when using finite element modeling to simulate and predict the occurrence of extra-axial hemorrhage.

Anatomically, three meningeal layers lie between the brain and skull: the dura mater, which is firmly attached to the skull, the arachnoid mater in the middle, and the pia mater, which adheres to the surface of the brain following the gyri and sulci. Together, the arachnoid and pia mater constitute the leptomeninges, or pia–arachnoid complex (PAC), consisting of cerebrospinal fluid (CSF), vasculature, arachnoid trabeculae, and the membranes themselves. Various techniques have been used to investigate the motion of the brain in response to the roughly rigid body motion of the skull including high-speed videography and replacement of a portion of the skull with a lucite calvarium (Pudenz and Shelden, 1946), flash X-ray cinematography (Hodgson et al., 1966; Gurdjian et al., 1968; Shatsky, 1973; Shatsky et al., 1974; Stalnaker et al., 1977), high-speed biplane X-ray and neutral density targets (Hardy et al., 2001, 2007; Zou et al., 2007), tagged magnetic resonance imaging (Bayly et al., 2005; Sabet et al., 2008; Feng et al., 2010), and most recently, magnetic resonance elastography (Badachhape et al., 2017) and three-dimensional digital sonomicrometry (Alshareef et al., 2017). Furthermore, finite element models have employed differing brain–skull boundary condition representations, including rigid attachment between the brain and skull, frictionless sliding, frictional contact, a layer(s) of fluid elements, a layer(s) of solid elements, or linear elastic connector elements between the outer brain and inner skull surfaces (Zhang et al., 2001, 2002; Kleiven and Hardy, 2002; Wittek and Omori, 2003; Cloots et al., 2008; Takhounts et al., 2008; Couper and Albermani, 2010; Coats et al., 2012; McAllister et al., 2012). However, few tissue mechanical studies and finite element models focus on definitive properties of the PAC with precision (Jin et al., 2006, 2007, 2011; Scott et al., 2015). Our lab recently found that linear elastic spring connectors imposed between the brain and skull provided good agreement between axial plane physical model brain–skull displacements (Ibrahim et al., 2010) and finite element model brain–skull displacements (Coats et al., 2012). Furthermore, this PAC representation predicted the occurrence of extra-axial hemorrhage in a rapid non-impact rotation model of TBI in porcine neonates with 80% sensitivity and 85% specificity based on the peak strain levels observed in the connector elements (Coats et al., 2012).

However, we have also shown that physiological and histopathological responses, clinical presentations, and behavioral outcomes are all dependent on the directional plane of rotational injury in our porcine TBI model (Eucker et al., 2011; Maltese, 2012; Sullivan et al., 2013), and similar phenomena have been noted in other studies in pigs (Smith et al., 2000; Browne et al., 2011), rats (Mychasiuk et al., 2016), non-human primates (Gennarelli et al., 1982, 1987), and humans (Pellman et al., 2003; Broglio et al., 2010; Stephens et al., 2016). In this communication, we build on our previous studies to investigate whether the response and properties of the neonatal porcine brain–skull interface are also dependent on the direction of rotation or region of the cranium, and if so, determine appropriate direction or region-specific boundary representations for finite element models of neonatal head rotation.

We measured brain–skull displacements from a series of sagittally transected piglet heads subjected to sagittal plane rotations, developed a finite element model mimicking the geometry of the sagittally transected piglet head, and determined a brain–skull boundary condition in the finite element model that matched finite element displacements to experimentally derived values. In addition, to investigate the effect of bridging vein behavior on mechanical tethering between the brain and skull, a range of bridging vein stress-stretch failure behavior was employed in the finite element model and the effect of varying bridging vein behavior on the brain–skull boundary condition was elucidated. We also compared our results to those reported previously in axial plane physical and finite element model transections of the neonatal pig (Ibrahim et al., 2010; Coats et al., 2012). The optimized sagittal brain–skull boundary condition may be used in future finite element models of the entire neonatal porcine head when sagittal plane rotations are simulated to find estimated elongations of the parasagittal bridging veins and determine the likelihood of extra-axial hemorrhage.

## Materials and Methods

### Physical Model

#### Construction

Using methods similar to those outlined in previous studies (Ibrahim et al., 2010; Coats et al., 2012; Sullivan et al., 2015), 3- to 5-day-old female Yorkshire piglets (*n* = 3) were used to construct physical transection models for the study of sagittal plane brain movements during rapid head rotation. Animal care and euthanasia procedures were approved by the University of Pennsylvania Institutional Animal Care and Use Committee. Briefly, each piglet was anesthetized with isoflurane, and then euthanized by administration of a lethal dose (150 mg/kg) of sodium pentobarbital. Immediately after euthanasia, the piglet was decapitated at the cervical spine, and all exterior soft tissue and the mandible were removed. A cutting plane was marked in the sagittal plane along the midline of the skull, and the skull was cut using a Dremel rotary tool with diamond wheel attachment with care taken to leave the meninges and brain intact. Once the skull was breached, the meninges and brain were sliced with a brain sectioning knife. The left side of the skull, meninges, and brain was discarded.

The right side of the transected head was embedded with the cut surface facing outwards in an aluminum pan with a 5" top inner diameter using polymethylmethacrylate (PMMA, Dentsply, York, PA, USA). The specimen-pan preparation was then attached to a custom aluminum canister *via* three set screws inserted through the side of the canister and aluminum pan, and into the curing PMMA. The skull edge was level with that of the aluminum canister. Preliminary tests were conducted, finding that brain temperature rose no higher than 37.1°C [below the average newborn piglet body temperature of 39°C (Lossec et al., 1998)], ensuring no tissue damage during PMMA curing.

**Figure 1:**
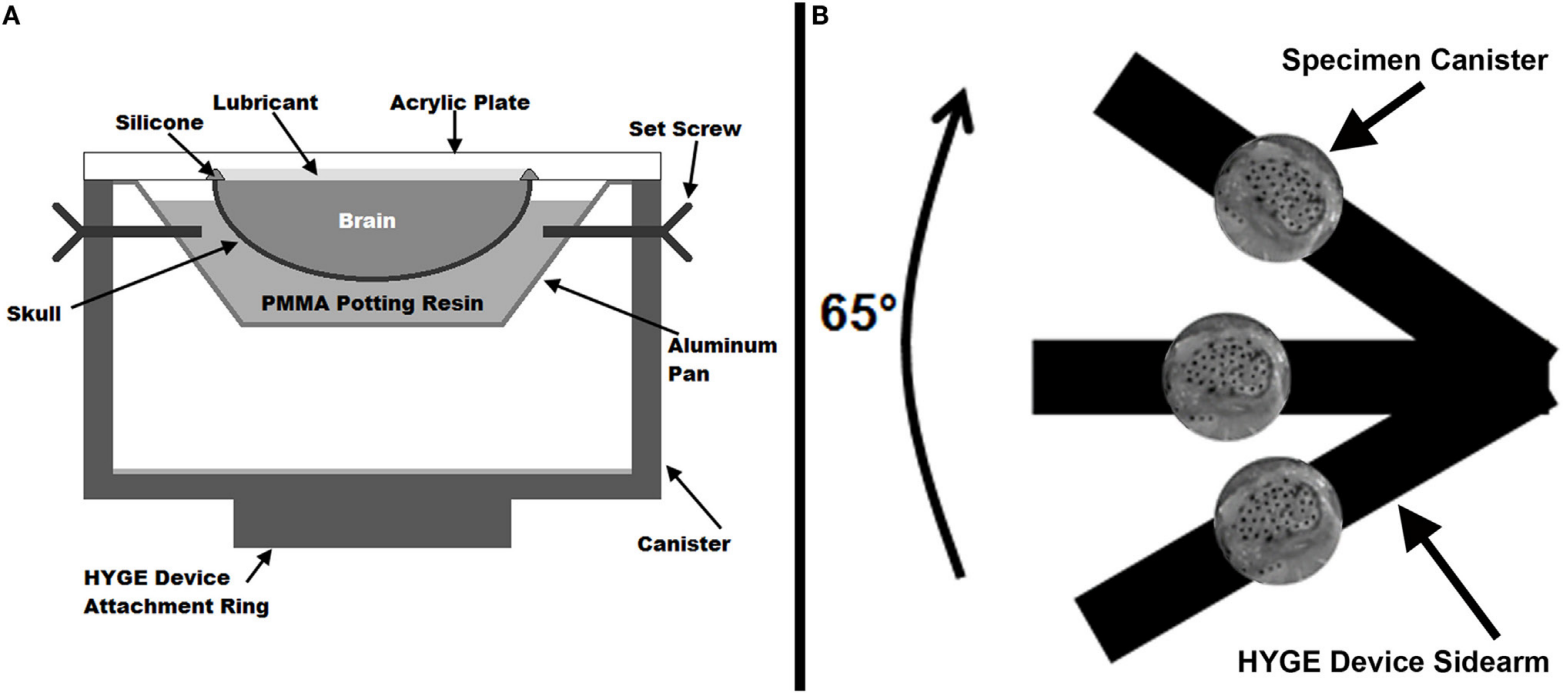
**(A)** Cross-sectional diagram of the physical sagittal plane transection canister configuration. **(B)** Schematic diagram (not to scale) of experimental setup on the HYGE device. The canister shows a representative example of the high-speed camera view of a sagittally transected specimen with India Ink marker dots.

Once the PMMA had hardened, 24–48 dots, ~2–3 mm in diameter, were applied to the exposed surface of the brain (India Ink, Speedball Art, Statesville, NC, USA). An additional 6–16 dots were placed on the skull and PMMA and used to assess rigid body motion. A border of clear silicone caulk was applied on top of the exposed skull edge to provide a seal around the brain and between the skull and a clear acrylic cover plate. Throughout the preceding steps of the preparation, the brain tissue was kept moist by periodic application of 1× phosphate-buffered saline on the cut surface. A thin layer of transparent lubricant (KY Brand, Skillman, NJ, USA) was applied to fill the space between the brain and the cover plate to minimize friction between the brain and the plate. Finally, the cover plate was secured to the canister ensuring no air bubbles were present within the lubricant, between the cover plate and specimen. All sagittal transection models (schematic, Figure 1A) were prepared and tested within 6 h of sacrifice.

#### Rotation

The canister assembly was mounted on one side arm of a custom linkage that converts linear motion generated by a HYGE device (Bendix Corporation, Southfield, MI, USA) into a 65° angular rotation (Raghupathi and Margulies, 2002), as shown in Figure 1B (schematic representation, not to scale). The center of rotation of the linkage was within the piglet cervical spine relative to the center of mass of the brain in the canister assembly. Each specimen was subjected to two consecutive rotations with peak velocities averaging 51.3 ± 1.2 rad/s (rotation 1) and 52.0 ± 1.7 rad/s (rotation 2). Experiments were filmed with a high-speed video camera (HG TH, Red Lake, Tallahassee, FL, USA) at 2,500 fps with a resolution of 320 × 480 (~0.41 mm/pixel). Angular velocity was measured by two angular rate sensors (ARS-06, Applied Technology Associates, Albuquerque, NM, USA) mounted to the linkage side arm, and recorded at 10,000 Hz using LabView software (National Instruments, Austin, TX, USA).

#### Image Processing

To enhance the visibility of the marker dots, image contrast and brightness were batch adjusted in Adobe Photoshop CS4 (San Jose, CA, USA) before analysis. A previously reported (Ibrahim et al., 2010; Sullivan et al., 2015) custom MATLAB script (The Mathworks, Natick, MA, USA) was used to filter the gray-scale images, and then isolate and track the marker dots frame by frame. In each frame, the centroid *x* and *y* pixel coordinates for each successfully segmented dot were defined in a global stationary reference system and recorded. To track individual markers across time, the nearest dot centroids within a 10-pixel distance of those in the preceding frame were assigned the same marker label.

#### Data Analysis

**Figure 2:**
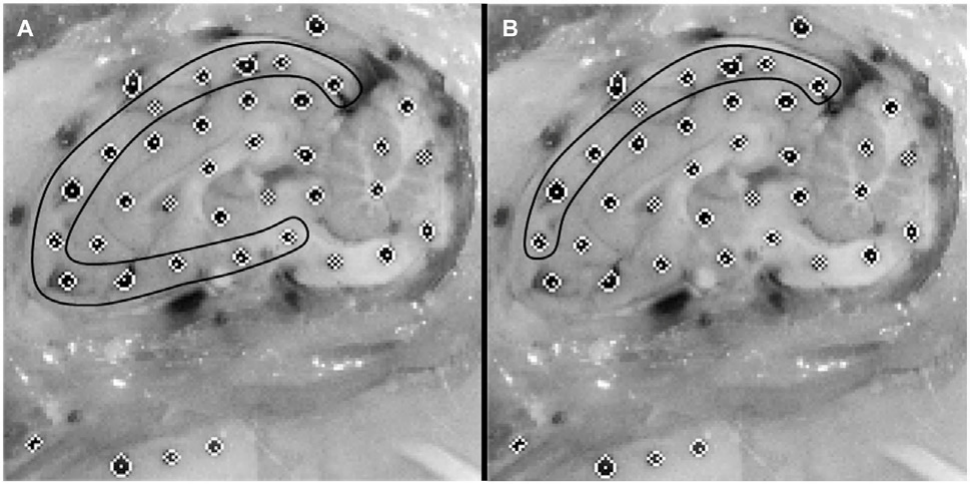
Example of an initial cropped video frame of a physical transection experiment. **(A)** Brain dots around whole cortex periphery are selected. **(B)** Superior-only brain dots are selected.

Dots on the periphery of the cut brain cerebrum surface were matched to the nearest rigid surface marker on the skull or PMMA to assess brain displacement relative to the rigid skull throughout the canister rotation (Figure 2). The peripheral brain dots were selected for analysis as they are nearest to the surface of the brain and, as such, would give the best estimate of brain surface displacement relative to the skull. This resulted in 13 brain-rigid dot pairs in the first specimen, 14 brain-rigid dot pairs in the second specimen, and 14 brain-rigid dot pairs in the third specimen. The distance between peripheral cortex brain dot centroids and their respective rigid dot centroids was calculated for each video frame from 50 frames before canister motion began until the frame in which the brain had stopped moving. For each brain-rigid dot pair, the calculated distances over the 50 frames before motion began were averaged to define a baseline distance for each brain-rigid dot pair. The baseline distance for each pair pre-motion was subtracted from the respective brain-rigid dot pair’s calculated distance in each frame to find the relative resultant displacements between brain and rigid dots in the sagittal plane over the 50 frames before motion and the duration of the rotation. The maximum relative displacement achieved by each brain-rigid dot pair during rotation was extracted over the experimental frames, while the maximum relative displacement found in each brain-rigid dot pair in the 50 frames before motion was extracted as an error measure correlating with the maximum experimental displacements.

The maximum experimental displacements and respective error measures for each brain-rigid dot pair were pooled across subjects for a total of 41 brain-rigid dot pairs. A series of Wilcoxon signed-rank tests were conducted to determine whether significant differences existed between the maximum displacements found during a rotation and their respective pre-motion errors, maximum displacements observed in the first and second rotations, and errors in the first and second rotations. Maximum displacements observed during the first rotations were also compared with those from other transection experiments performed previously in the axial plane at similar angular velocities (Ibrahim et al., 2010; Coats et al., 2012), again using a Wilcoxon signed-rank test. For the axial plane transection experiments, brain distortion was reported previously. Here, we report brain–skull displacement for the first time.

### Finite Element Modeling

#### Construction and Simulation

**Figure 3:**
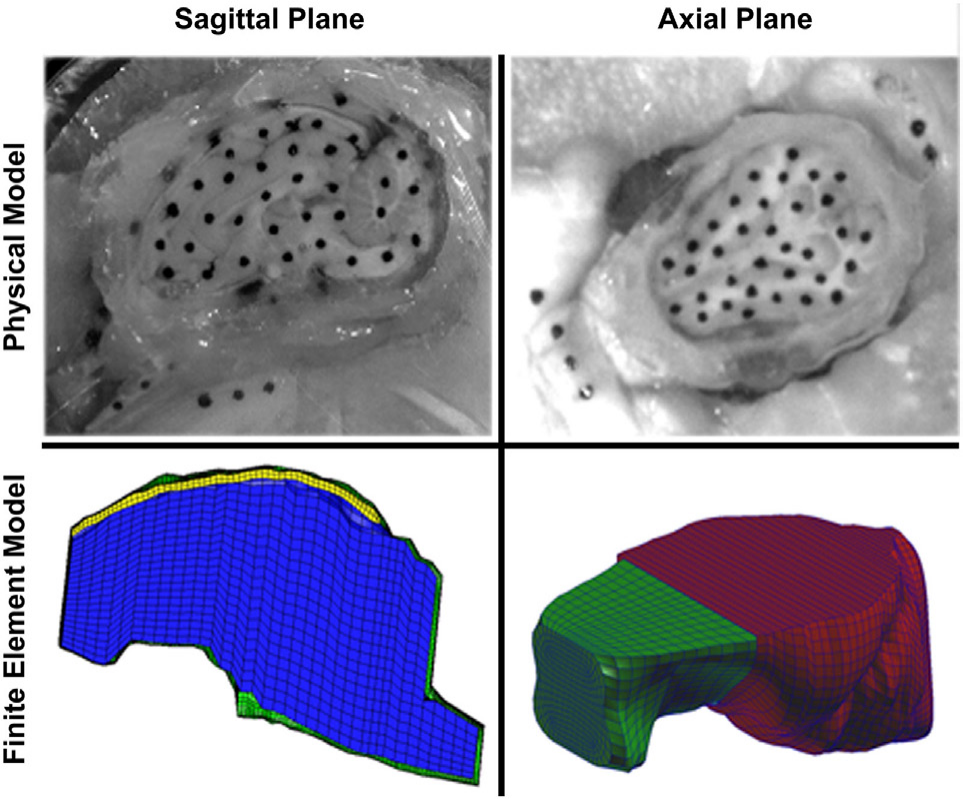
Comparison of sagittal (left) and axial (right) transection physical models (top) and finite element models (bottom). In the sagittal transection finite element model, the brain is represented by blue elements, falx is represented by yellow elements, and the skull is represented by green elements. In the axial transection finite element model, the brain is represented by green elements, and the skull (and cover plate) is represented by red elements. Note the more symmetric shape of the axial cross-section compared with the sagittal cross-section.

To create finite element model geometry similar to the sagittal transection physical configuration, we modified a previously reported piglet finite element model with stable brain mesh convergence (Coats et al., 2012; Sullivan et al., 2015). To mimic the sagittal transection experimental configuration, the converged 3- to 5-day-old piglet model was transected at the sagittal midline, retaining the right side of the model. The resulting transection model (Figure 3, bottom left) had 6,509 linear hexahedral brain elements, 732 linear wedge falx elements, and 8,858 rigid shell skull elements. The frictionless boundary condition between the transected surface of the brain and the acrylic cover plate was replicated by imposing a kinematic coupling constraint on the corresponding finite element model surface such that the brain nodes on the cut surface were free to translate in the cut plane, but were prevented from out of plane deformations.

The brain elements were defined as a homogenous, isotropic, and non-linear hyperelastic material according to a first-order Ogden strain energy density function (Eq. 1):

(1) $$W=\frac{2\mu (t)}{\alpha^{2}}(\lambda_{1}^{\alpha }+\lambda_{2}^{\alpha }+\lambda_{3}^{\alpha }-3),$$

where the λs are the principal stretch ratios, α is a non-linear tissue-specific parameter dependent on strain magnitude, and μ(*t*) is the brain shear relaxation modulus. The brain shear relaxation viscoelastic response was modeled using a two-term Prony series function (Eq. 2):

(2) $$\mu (t)=\mu_{0}\left(1-\sum_{i=1}^{2}C_{i}(1-e^{-t/\tau_{i}})\right),$$

where μ_0_ is the brain shear modulus, *C_i_* are the relaxation moduli, and τ*_i_* are time constants. The relaxation moduli and time constants were selected based on those published in our previous study investigating high strain deformation of porcine brain (Prange and Margulies, 2002). Brain shear modulus was selected as the low end of an optimized range of 3- to 5-day-old piglet brain shear moduli (553 Pa) determined from reverse engineering brain tissue strains measured in prior *in situ* axial transection experiments at higher velocities (121 rad/s) to the same porcine finite element model (Sullivan et al., 2015). This value is similar to previously reported measures of *in vitro* shear modulus (526.9 Pa) (Prange and Margulies, 2002) and *in situ* shear modulus (541 Pa) (Bradfield, 2013). Utilizing reported values for elastic modulus and average specimen dimensions of adult human dura reported by Galford and McElhaney, and values for stiffness of fetal human dura reported by Bylski et al., it can be approximated that the stiffness of adult human dura is twice that of fetal human dura (Galford and McElhaney, 1970; Bylski et al., 1986; Coats et al., 2012). Assuming this relationship holds for elastic modulus and that properties are similar across species, the falx was defined as an isotropic, linear elastic material with elastic modulus half that of adult human dura (Coats et al., 2012). The skull was modeled as a rigid body. All material properties and constants are reported in Table 1.

**Table 1:** Material properties used in the sagittally transected porcine head finite element model.

Tissue	Material property	Reference
Brain	μ_0_ = 553 Pa	Sullivan et al. (2015)
	α = 0.01	Prange and Margulies (2002)
	*C*_1_ = 0.3322	Prange and Margulies (2002)
	*C*_2_ = 0.3890	Prange and Margulies (2002)
	τ_1_ = 2.9572 s	Prange and Margulies (2002)
	τ_2_ = 0.1813 s	Prange and Margulies (2002)
	ρ = 1.04 g/cm^3^	Sullivan et al. (2015)
	ν = 0.49999	Sullivan et al. (2015)
Falx	ρ = 1.13 g/cm^3^	Coats et al. (2012)
	*E* = 15 MPa	Sullivan et al. (2015)
	ν = 0.45	Coats et al. (2012)
General brain–skull connectors	*k* = 34.6–4,000 N/m	N/A

A previous study using the axial transection and full head configurations of this newborn porcine computational model examined the boundary condition between the brain and skull during axial direction rotations of separate, previously reported axial plane physical transections (Ibrahim et al., 2010), and found that linear elastic spring connectors linking every brain surface node to the nearest skull node provided an approximate combined response of the PAC and CSF such that brain tissue strain and brain–skull displacement were similar between physical and finite element estimations (Coats et al., 2012). Not including the sagittal cut plane, the sagittally transected model had 1,398 brain surface nodes. Each brain surface node was matched to the nearest node on the inner surface of the skull or falx. Eleven of these node pairings were reserved for elements representing bridging veins, according to the average number of bridging veins found per hemisphere in a study of human adult cadavers as these data are unavailable for the pig (Han et al., 2007), leaving 1,387 two-dimensional linear elastic connector elements to define the boundary condition between the brain and skull. We hypothesize that brain–skull displacement in the axial plane may differ from that in the sagittal plane, and one objective was to optimize the elastic stiffness of the connectors based on measured brain–skull displacements from the physical transection experiments. A total of 15 different elastic stiffnesses ranging from 34.6 to 4,000 N/m, including the stiffness of 3,460 N/m used for axial plane transection models, for the general brain connectors (listed explicitly in Section “Results”) were simulated in all six sagittal transection simulations (*n* = 3 piglets × 2 rotations/piglet). Potential contact between the brain and skull or brain and falx that was not prevented by the general connector or bridging vein connector elements was governed by a friction surface interaction with a coefficient of 0.2 (Coats et al., 2012). The superior surface of the falx was rigidly tied to the inner surface of the skull (Coats et al., 2012).

In axial and coronal plane rotations, the falx cerebri may prevent lateral brain movement near the superior cortical surface (Pudenz and Shelden, 1946; Hardy et al., 2007; Sabet et al., 2008). Conversely, in sagittal plane rotations, the falx cerebri does little to prevent brain–skull relative displacement and resulting bridging vein elongation, thus it is important to include bridging vein elements to account for their potential tethering influence at the brain–skull boundary. The 11 bridging veins were chosen from brain nodes that connected to nodes on the inner surface of the falx as the superior sagittal sinus, into which the bridging veins drain, is housed inside the falx. To assign bridging vein element locations, the length of the finite element model falx was divided from anterior to posterior, into two short segments followed by two longer segments comprising 20, 20, 30, and 30% of the total length, respectively. The first segment contained five bridging veins and the third contained six bridging veins, while the second and fourth segments had no bridging veins, matching the clustering observed in adult cadavers, again, as these data are unavailable for the pig (Han et al., 2007). Bridging vein connection points were selected such that they were evenly distributed in an anterior–posterior sense within their tributary falx segments and midline connection points were excluded as the bridging veins enter from the hemispheres more laterally. Together with their corresponding brain connection points, these brain–falx node pairs comprised the 11 bridging vein elements in this sagittal transection model, and the initial length of each bridging vein element was found based on the undisturbed positions of their respective brain and falx nodes.

The bridging veins span the distance from the brain to the superior sagittal sinus through the meninges and CSF. They are flaccid and cannot bear compressive loads along their longitudinal axis. Thus, the primary loading modality when stretched between their attachment points is assumed to be longitudinal tension. Therefore, bridging veins were represented by two-dimensional non-linear axial connector elements, with force–displacement curves prescribed according to averaged post-cyclic porcine newborn stress–stretch curves obtained in a previous study (Pasquesi and Margulies, 2017), and no load upon compression. Briefly, newborn porcine parasagittal bridging veins were subjected to one of three protocols: elongation to failure at a high stretch rate, elongation to failure at a low stretch rate, or cyclic loading followed by elongation to failure at a rate similar to, but higher than the low rate tests. Stress-stretch failure behavior and mechanical properties were evaluated and compared across protocols. Post-cyclic loading stress-stretch curves displayed a longer low stress toe region than low and high rate curves, while high rate testing resulted in the stiffest behavior measurements. Thus, post-cyclic loading stress-stretch failure curves were chosen as a worst-case scenario in terms of the ability of the bridging veins to provide any mechanical resistance to displacement between the brain and skull. After optimization of the brain–skull boundary condition with post-cyclic loading bridging vein failure behavior (described in Section “Data Analysis”), a simulation was performed using high rate bridging vein failure behavior to determine whether the optimized brain–skull boundary condition was sensitive to the span of bridging vein behaviors measured previously (Pasquesi and Margulies, 2017). For force calculations, all bridging vein elements were assumed to have the same cross-sectional area, assigned as the average cross-sectional area of all porcine newborn bridging veins tested previously (Pasquesi and Margulies, 2017), and displacements were calculated based on individual initial bridging vein element lengths.

To match physical transection ink dots to finite element model node locations, every brain perimeter and rigid ink dot centroid used in analysis of brain–skull displacement in the physical transection experiments was matched to a corresponding node in the finite element model by overlaying images of segmented dots in the physical experiment with an image of the sagittal cut surface of the finite element model in Adobe Photoshop CS4 (San Jose, CA, USA). If an ink dot centroid did not align exactly with a sagittal plane finite element node, up to four surrounding nodes were assigned to that brain or rigid centroid such that their coordinates may average to the approximate location of the ink dot. The brain and skull nodes corresponding to brain and rigid ink dot coordinates were tracked throughout the finite element model simulation and subsequently analyzed for maximum displacement for comparison with values determined by the physical transection models (see Data Analysis). These displacements were used to refine the general linear elastic connector boundary condition by adjustment of the assigned elastic stiffness such that finite element maximum displacements were representative of those observed in the physical experiments.

The angular velocity–time histories from the physical transection experiments were used as load inputs for the corresponding finite element model simulations, filtered at CFC 200 to ensure sufficient removal of high frequency artifacts for simulation, while maintaining the shape of the velocity–time curve and peak angular velocities. All simulations were conducted in ABAQUS Explicit version 6.11 (Simulia, Providence, RI, USA) with double precision. To eliminate excessive brain deformation and negative element volumes, distortion and enhanced hourglass control were activated for brain elements. The mass of the intact (non-transected) brain totaled 44.6 g in the finite element model (Coats et al., 2012; Maltese, 2012; Sullivan et al., 2015) and was not scaled by experiment as individual brain masses from the physical transection experiments could not be recorded.

#### Data Analysis

For each general connector stiffness tested, the maximum relative displacement across time between brain and skull nodes corresponding to physical experiment brain and rigid ink dots was extracted. The maximum relative displacements were pooled across all simulations and compared with the corresponding physical model maximum relative displacements using a linear regression with intercept at 0:

(3) $$d_{\text{FEM}}=a*d_{\text{EXP}},$$

where *a* is the slope of the linear regression, *d*_FEM_ is the maximum finite element model displacement, and *d*_EXP_ is the maximum physical model experimental displacement. If the 95% confidence interval for slope contained 1, the stiffness was deemed to be a good choice for boundary condition between the brain and skull. As several of the tested stiffnesses fit this criterion, the same linear regression analysis was repeated on brain–skull node and brain-rigid dot pairs located on the superior surface of the brain only (Figure 2), since the superior surface displacement determines bridging vein elongation. The stiffness that resulted in slope values within the narrowest range of 1 for both all node and dot pairs and superior-only node and dot pairs with 95% confidence intervals for slope including 1, was chosen as the optimal boundary condition stiffness for future analyses.

In a final analysis, to determine the sensitivity of the optimized brain–skull boundary condition to the span of bridging vein behaviors measured previously, the averaged newborn porcine high rate bridging vein stress–stretch curve and resulting force–displacement curves were applied to the bridging vein elements. The resulting slope values between finite element and experimental displacements for all brain–skull/rigid dot pairs and superior-only brain–skull/rigid dot pairs were compared between the use of post-cyclic bridging vein curves and high rate bridging vein curves.

## Results

### Physical Model

#### Measurement Error

No significant differences were noted in brain–skull displacement measurement error between the first and second rotations when pooled across subjects, as described in Section “Data Analysis.” To calculate average digitization error, brain–skull displacement measurement errors were averaged across all dot pairs in each rotational experiment and were further averaged across all subjects for first rotations, second rotations, and all experiments combined (Table 2). The overall average brain–skull displacement measurement error was 0.31 ± 0.23 mm, which corresponded to <1 pixel difference in the video frames.

**Table 2:** Peak angular velocities, peak angular accelerations, peak angular decelerations, average maximum brain–skull displacements during motion, and average brain–skull displacement errors for all cortex periphery brain-rigid dot pairs are shown for each transection experiment.

Animal	Rotation number	Peak angular velocity (rad/s)	Peak angular acceleration (krad/s^2^)	Peak angular deceleration (krad/s^2^)	Average of maximum brain–skull displacements (mm)	Average brain–skull displacement error (mm)
1	1	52	9.6	6.8	1.00 ± 0.41	0.32 ± 0.16
1	2	51	10.3	7.4	1.07 ± 0.40	0.28 ± 0.17
2	1	50	8.4	8.6	0.63 ± 0.36	0.28 ± 0.19
2	2	51	9.5	8.1	0.68 ± 0.29	0.31 ± 0.37
3	1	52	6.2	9.0	0.58 ± 0.27	0.33 ± 0.29
3	2	54	7.7	11.2	0.66 ± 0.26	0.31 ± 0.21

Rotation 1 avg.		51.3 ± 1.2	8.1 ± 1.7	8.1 ± 1.2	0.73 ± 0.39^†^	0.31 ± 0.21*
Rotation 2 avg.		52.0 ± 1.7	9.2 ± 1.3	8.9 ± 2.0	0.80 ± 0.37^‡^	0.30 ± 0.25*

Overall avg.		51.7 ± 1.4	8.6 ± 1.5	8.5 ± 1.5	0.76 ± 0.38	0.31 ± 0.23

*Values are pooled among first and second rotations, as well as across all experiments*.

Significant differences between groups (*p* < 0.01) are indicated by different symbols (*, ^‡^, ^†^).

#### Brain–Skull Displacement

**Figure 4:**
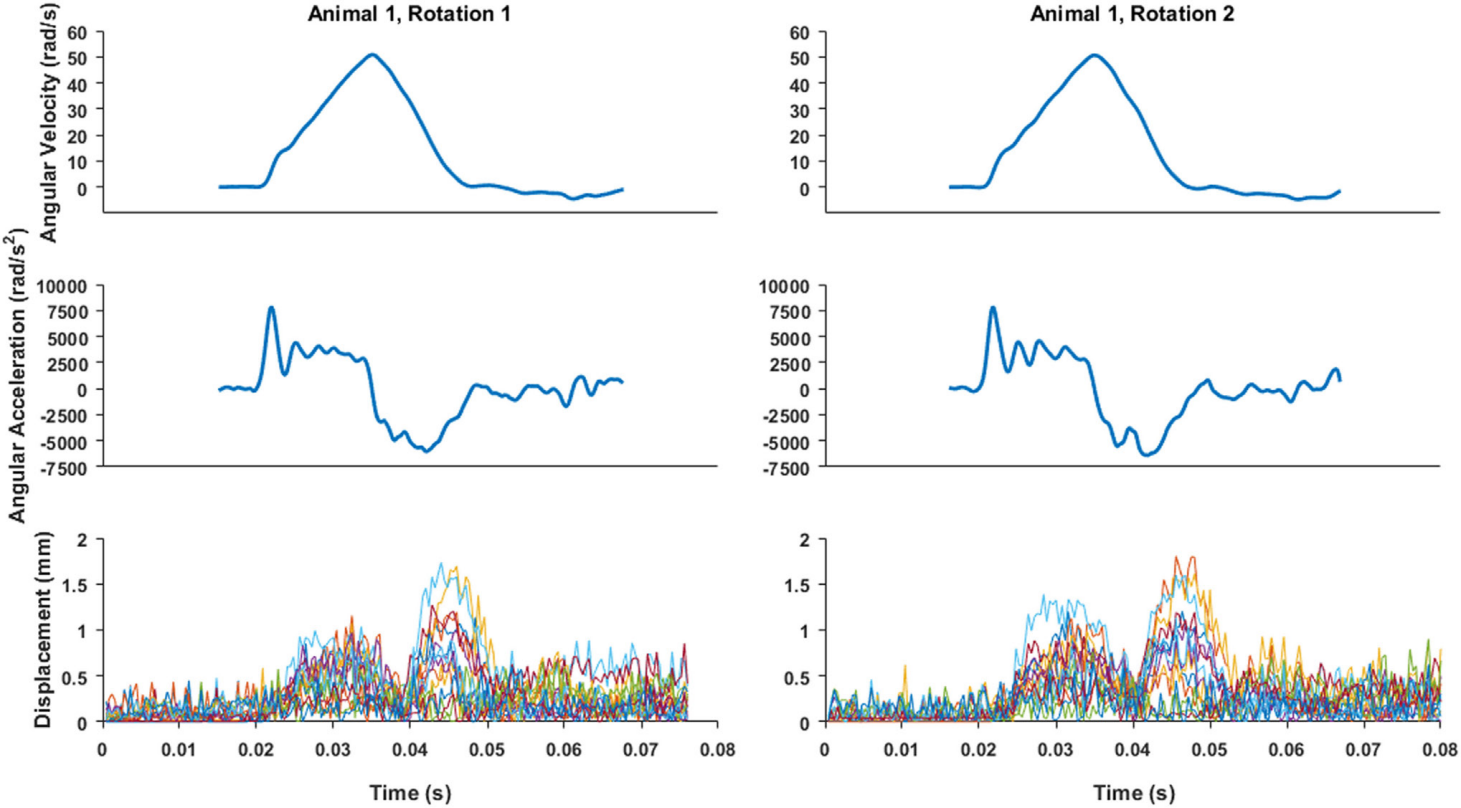
An example of angular velocity and angular acceleration time histories for Animal 1 transection rotations and the associated brain-rigid dot displacement for 13 dot pairs around the whole cortex periphery. Pre-motion frames include time points up to 0.02 s. While angular velocity and acceleration plots are on the same time scale as the displacement plot, the velocity and video camera data recording triggers were not simultaneous, thus the beginning of the angular velocity and acceleration time histories were aligned such that the time point at which angular velocity began monotonically increasing toward its peak was translated to 0.02 s on the brain–skull displacement plot.

Brain–skull displacement appears to peak at two time points during the angular velocity pulse, with maximal values at the end of rotation deceleration (Figure 4). Average maximum relative displacements between brain and rigid dots in each rotational experiment, pooled across first rotations, pooled across second rotations, and across all experiments combined, are shown in Table 2. Maximum relative displacements in each brain-rigid dot pair during the first and second rotations were significantly greater than their respective error measurements (*p* < 0.000001 for both tests). Thus, we conclude that there was measurable relative translation between the brain and skull during rotational events. Furthermore, the maximum displacements in each brain-rigid dot pair were greater in the second rotation than in the first rotation (*p* = 0.0094). Coupled with our previous finding that the brain–skull displacement error measures were not different between the first and second rotations, we conclude that translation between the brain and skull is greater in the second rotation than the first rotation, indicating possible damage to the tethering leptomeninges between the brain and skull had occurred during the first rotation. However, the difference in average maximum relative displacements between the first and second rotations was less than our measured error level of 0.31 mm. Thus, the finding that more translation between the brain and skull occurred during the second rotation may be of negligible importance, but additional consecutive rotations (beyond 2) should be performed in future transection preparations to determine if increasingly greater relative displacements occur with sequential rotations.

**Figure 5:**
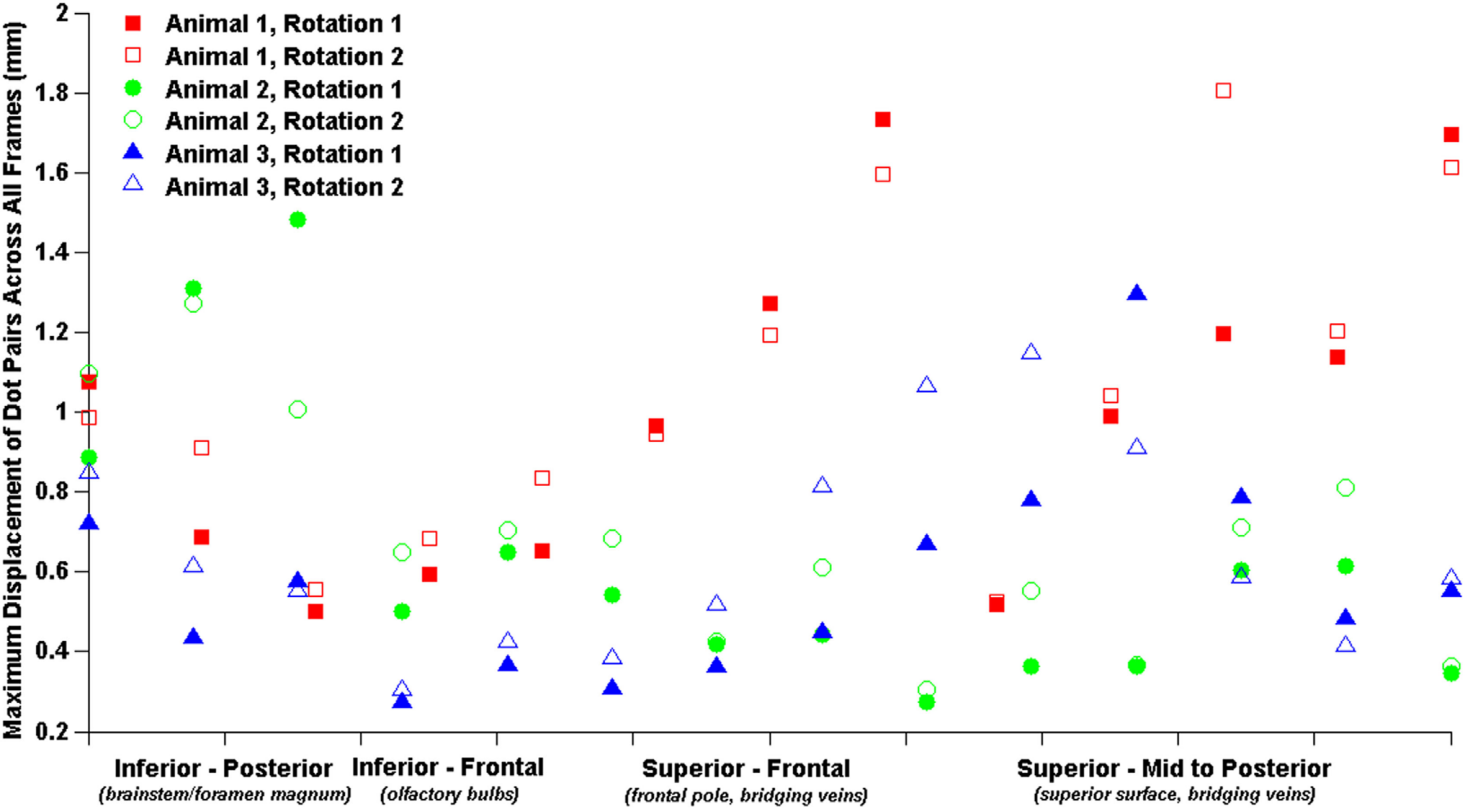
Brain–skull/rigid dot maximum sagittal physical transection displacements around cerebrum periphery.

The relatively coarse resolution of the physical model ink dots prevents detailed regional analysis of brain–skull displacements around the sagittal transection perimeter. Nonetheless, we plotted brain–skull/rigid dot pair displacements with brain dots around the periphery of the transected cerebrum from inferior–posterior to superior–posterior and found increased maximum displacements near the brainstem/foramen magnum, and near the superior surface (Figure 5). Future studies may attempt to obtain higher resolution high-speed videos for better analysis of regional brain–skull displacement in the sagittal plane, and refinement of finite element model boundary conditions.

**Figure 6:**
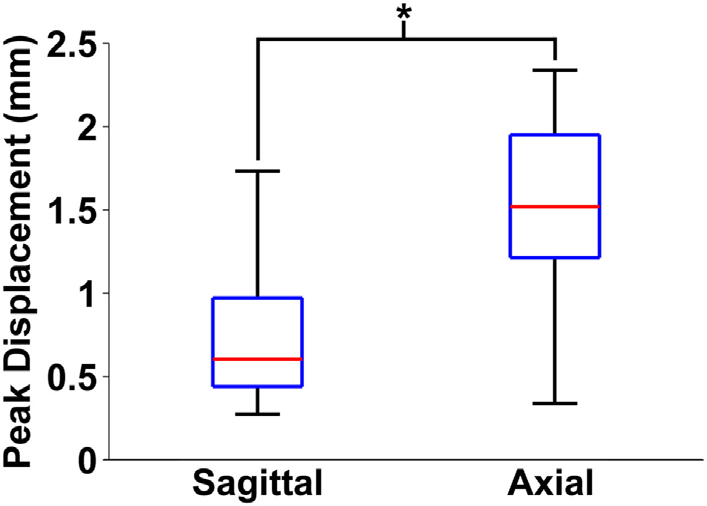
Box and whiskers plot of peak brain–skull displacements measured in first rotation sagittal transections from this study and first rotation axial transections detailed in previous work. The middle line in each box indicates median value, while the bottom and top edges of the box are the 25th and 75th percentile values. Finally, the whiskers extend to the most extreme high and low values in each data set. Axial plane brain–skull displacements were significantly higher than sagittal plane brain–skull displacements (*p < 0.000001).

Finally, maximum relative displacements that occurred during the first rotation were also compared with those analyzed from experiments of axially transected piglet heads rotated in the axial plane at similar levels of peak angular velocity (Ibrahim et al., 2010; Coats et al., 2012). Average maximum displacements recorded in each axial transection experiment are shown in Table 3. Maximum displacements in the axial transection experiments were significantly higher than those recorded in the sagittal transections in this study (*p* < 0.000001, Figure 6).

**Table 3:** Previously conducted newborn piglet axial transection experiment peak angular velocities, peak angular accelerations, peak angular decelerations, and average maximum brain–skull displacements around the entire axially transected cortex periphery.

Animal	Peak angular velocity (rad/s)	Peak angular acceleration (krad/s^2^)	Peak angular deceleration (krad/s^2^)	Average of maximum brain–skull displacements (mm)
Axial—1	52	6.2	6.9	1.71 ± 0.40
Axial—2	53	6.7	6.3	1.50 ± 0.56
Axial—3	54	5.5	6.6	1.31 ± 0.56

Overall	53.0 ± 1.0	6.1 ± 0.6	6.6 ± 0.3	1.50 ± 0.53

*These axial transection experiments were conducted at peak velocities (53.0 ± 1.0 rad/s) similar to those used in our sagittal transection experiments (51.3 ± 1.2 rad/s) and had no prior load history. Kinematic data (and brain parenchyma distortion) were previously reported (Ibrahim et al., 2010), and while these brain–skull displacements were used in another computational study examining the occurrence of extra-axial hemorrhage (Coats et al., 2012), this is the first time they are being reported explicitly*.

### Finite Element Model Optimization

Using both all peripheral brain–skull/rigid dot pairs and superior-only brain–skull/rigid dot pairs, a two order of magnitude range of general linear elastic connector stiffnesses was analyzed, and several yielded 95% confidence intervals for slope that included a value of 1 between the measured and simulated boundary displacements (Table 4). Employing the general connector stiffness used in axial transections of this model (3,460 N/m) in the sagittal transection model resulted in brain–skull displacements that were smaller than those measured in our sagittal transection physical models. The optimal stiffness for the sagittal transection model was 46.133 N/m because slopes for all dot pairs and superior-only pairs were within the narrowest range of 1 (Table 4). The general linear connector boundary condition in the finite element model was assigned this value. When high rate bridging vein properties were implemented with this optimized general connector stiffness, very little difference in slope between finite element and experimental displacements was observed (Table 4), suggesting that in comparison with other structures in the PAC, the bridging veins may not contribute much mechanical resistance to translation between the brain and skull. Of note, no bridging vein stretch exceeded the average ultimate stretch ratio of newborn porcine bridging veins (Pasquesi and Margulies, 2017).

**Table 4:** All stiffness values tested for general brain–skull/falx connectors in the sagittally transected finite element model, and corresponding slopes between finite element and physical transection experiment brain–skull displacements in Eq. 3 over all brain dots, *a*_all_, and superior-only brain dots *a*_sup_.

Stiffness (N/m)	*a*_all_	*a*_sup_
4,000	0.769 (0.684, 0.854)	0.665 (0.582, 0.749)
3,750	0.769 (0.684, 0.855)	0.666 (0.582, 0.749)
3,460^a^	0.770 (0.685, 0.855)	0.666 (0.582, 0.750)
3,250	0.770 (0.685, 0.855)	0.666 (0.583, 0.750)
3,000	0.771 (0.686, 0.856)	0.667 (0.583, 0.750)
2,595	0.772 (0.687, 0.857)	0.668 (0.584, 0.751)
2,000	0.774 (0.700, 0.859)	0.669 (0.586, 0.753)
1,730	0.775 (0.690, 0.861)	0.670 (0.587, 0.754)
692	0.789 (0.703, 0.875)	0.678 (0.596, 0.761)
346	0.812 (0.724, 0.899)	0.687 (0.608, 0.766)
138.4	0.930 (0.826, 1.034)	0.784 (0.695, 0.874)
69.2^b^	1.002 (0.879, 1.125)	0.883 (0.753, 1.014)
46.133^b^	1.082 (0.939, 1.226)	0.984 (0.8230, 1.144)
46.133^b^^,^^c^	1.081 (0.938, 1.225)	0.981 (0.821, 1.142)
34.6^b^	1.149 (0.990, 1.308)	1.056 (0.874, 1.239)

*95% Confidence intervals for slope are indicated in parentheses to the right of the derived slope value*.

*^a^The general connector stiffness used in previous porcine finite element models, optimized to axial brain–skull displacements (Ibrahim et al., 2010; Coats et al., 2012)*.

*^b^Stiffnesses for which the 95% confidence intervals for both a_all_ and a_sup_ contain 1*.

*^c^Finite element simulations were run with high rate bridging vein properties*.

*The optimal general connector stiffness chosen for further analysis is 46.133 N/m as the slopes for all brain–skull dot pairs and superior-only brain–skull dot pairs are within the narrowest range of 1*.

## Discussion

In this study, we measured the sagittal plane brain–skull displacement of the newborn porcine head when subjected to sagittal plane rotations, and provided an appropriate brain–skull boundary condition for finite element modeling of sagittal plane head rotations in the porcine newborn. We observed significant brain–skull relative displacements in an *in situ* model with sagittal plane rotations at peak velocities of ~52 rad/s. Brain–skull displacements during head rotations are direction dependent with those measured in the sagittal plane (0.73 ± 0.39 mm, rotation 1) in this study significantly lower than those measured in the axial plane (1.50 ± 0.53 mm). Interestingly, the brain–skull boundary condition prescribed to match respective physical transection experimental brain–skull displacements was far less stiff (46.133 N/m) in a sagittally transected finite element model of the porcine newborn head than that in a corresponding axial transection finite element model (3,460 N/m). These data will inform future full head finite element model simulations of rotational injury by providing a validated boundary condition for sagittal plane motion.

Measurement errors may be attributable to inconsistent lighting as the frame rate of the high-speed camera was faster than the frequency of ambient light in the laboratory, or small frame-to-frame discrepancies with dot edge detection. Regardless, measurement error averaged slightly less than one pixel distance (~0.31 mm versus ~0.41 mm/pixel) and was of a similar level as previous studies utilizing high-speed biplanar X-ray and neutral density targets in human cadavers (Hardy et al., 2001, 2007), in which SDs of the distance between skull markers ranged between 0.12 and 0.46 mm. Paired Wilcoxon signed-rank tests consistently showed that maximum brain-rigid dot displacements during rotation were higher than their respective error measures. Additional Wilcoxon signed-rank tests revealed that the maximum relative displacements between brain-rigid dots in the second series of rotations were higher than those in the first set of rotations, indicating that some structural damage may have occurred as a result of the first rotation. However, the difference in maximum displacements between the first and second rotations was less than our measurement error, rendering this finding statistically significant, but of low importance. While peak angular velocities and accelerations were often slightly higher in the second rotation of a given specimen than the first, it is also notable that Figure 4 shows small persistent increased displacements post-motion after an initial rotational event, possibly indicating damage to the brain–skull boundary (Hardy et al., 2007). It is unknown whether the slight increases in peak angular velocities and accelerations in the second series of rotations were a causative factor in the higher values measured in the second series of rotations. Future studies may examine more than two consecutive rotations to the same load level to investigate if maximum brain–skull displacements get progressively larger with an increasing number of rotations, such that they are greater than measurement error. Findings from these proposed studies would provide valuable information for modeling the brain–skull boundary condition in incidences of shaking or repeated insults.

We observed that brain–skull interface displacements vary with head rotation direction. Connector element density (connecters per square millimeter of brain surface area) was slightly less in the sagittal plane transection (0.37 connectors/mm^2^) than in the axial plane transection (0.40 connectors/mm^2^). Thus, we postulate that the irregular geometry of the sagittal cross-section results in smaller brain–skull displacements than in the axially sectioned brain and skull (Figure 3). Because the irregular geometry restricts motion of the brain, the geometric boundary dominates the brain–skull motion response in the sagittal transection and a lower connector stiffness (46.133 N/m) was required than in the axial section (3,460 N/m) (Coats et al., 2012). Specifically, the axial plane cross-section is smoother and rounder, with few impediments to movement (Figure 3). By contrast, the sagittal brain–skull boundaries near the cerebellum, brainstem, inferior frontal boundary near the porcine olfactory bulbs, and smooth superior surface may each uniquely and naturally restrain (possibly near the cerebellum, brainstem, and olfactory bulbs) or facilitate (possibly near the cerebellum or superior cerebrum surface) motion of the brain relative to the skull.

Our results suggest that bridging veins do not dominate the mechanical tethering between the brain and skull, whereas other structures may play a larger role. Few material property tests have examined the PAC, but the available data reveal that normal traction (Jin et al., 2007) and transverse shear (Jin et al., 2011) moduli of the bovine PAC, where arachnoid trabeculae determine the mechanical response are lower than the longitudinal bridging vein elastic moduli measured for both pigs and humans (Pasquesi and Margulies, 2017). On the other hand, the elastic modulus of the bovine PAC under in-plane tension (PAC, 6–40 MPa) (Jin et al., 2006) is similar to that of porcine and human bridging vein longitudinal elastic moduli (22–49 MPa) (Pasquesi and Margulies, 2017). It is likely that the PAC is loaded in a combination of normal traction, transverse shear, and in-plane tension during rapid rotations of the head, while it is likely that the bridging veins are loaded primarily in longitudinal tension between their attachment points. The interaction of these different PAC loading modalities with longitudinal tension of the bridging veins is unknown and should be investigated in the future. Furthermore, a microstructural imaging study of the neonatal porcine PAC revealed high regional variability in arachnoid trabeculae and subarachnoid vasculature volume fraction, indicating that PAC material properties vary across the brain surface (Scott and Coats, 2015). Our relatively coarse physical model ink dot resolution precluded a more detailed regional analysis in this study. While a few microscale finite element models have begun to address the individual and combined components of the PAC (Ma et al., 2008; Zoghi-Moghadam and Sadegh, 2009; Scott et al., 2015), only one study developed a corresponding macroscale PAC representation based on regional and interindividual PAC structural distributions (Scott and Coats, 2015), validated in a full head model against the presence of hemorrhage (Scott et al., 2015). While some material properties of the PAC representative elements were based on literature-reported values (Jin et al., 2006, 2007, 2011), the model response was not validated against *in situ* brain–skull displacements (Scott et al., 2015). Future combining of representations of the PAC with measured material properties, biofidelic regional and interindividual structural density ranges, and observed *in situ* brain–skull displacements may provide an even more biofidelic brain–skull boundary condition in finite element models. In addition, in all material property and finite element studies, the PAC is assumed to be transversely isotropic, but only in-plane tension tests have confirmed this along both the sagittal and coronal planes (Jin et al., 2006). Future studies should investigate the transverse shear properties of the PAC by loading in the approximate directions of the sagittal and coronal planes.

### Limitations

The presented studies include several limitations. First, despite a modest cohort size and limited statistical power, we found significant differences in brain–skull displacement between the first and second consecutive rotation experiments when pooling all brain-rigid dot pairs across specimens. Previous studies of axial plane transection preparations have relied on similar sample sizes when investigating brain parenchyma strain (Ibrahim et al., 2010; Bradfield, 2013; Sullivan et al., 2015). Nevertheless, future studies should utilize a larger sample size to confirm distinct differences in brain–skull displacements between consecutive rotational insults and directional planes. Second, it was impossible to capture brain–skull displacements orthogonal to the sagittal plane of rotation (and axial plane of rotation for prior axial studies) in the physical model transections. However, we assume any orthogonal displacements are likely minimal compared with those within the plane of rotation. Third, physical model construction may have caused structural alterations to the transected cranial contents. Most notable alterations include transection of the falx cerebri, restriction of motion of the brainstem through the foramen magnum and the probable draining of CSF. The neonatal porcine falx is not nearly as prominent a structure as it is in the human cranium and is difficult to see in the transected specimens. While care was taken to ensure physical transection was performed at the sagittal midline, whether portions of the falx remained in the tested specimen were not directly measured. The effect of the transection at the sagittal midline on bridging vein connections at the dura is unknown, although these connection points are typically slightly lateral to the midline and it is believed that these connections were preserved in the experimental setup. The physical experimental setup necessitated that the edge of the foramen magnum be embedded in potting media, restricting motion of the brainstem through the foramen magnum. Because this motion was restricted, we may be underestimating the motion of the surface of the brain relative to the skull, but the true effect of sealing the foramen magnum is unknown. It is notable that a similar restriction was prescribed in the associated finite element model. Care was taken to preserve the mechanical integrity of the brain, skull, and interfacing tissues, to fill the structures with buffered saline during preparation, and ensure that no air bubbles were observed within the lubricant between the specimen and acrylic cover plate as the presence of gas within the specimen may decouple the brain from the skull and permit larger relative motion between the brain and skull (Hardy et al., 2007). The pressures within the transected skull specimens were not measured. It is unknown how any pressure differences may have contributed to the measured brain–skull displacements, and what affect this may have had on the finite element model boundary condition determination. Fourth, despite image filtering, coarse resolution of ink dots combined with coarse video image resolution and pixel-wise dot segmentation resulted in relatively coarse measurements of dot displacement. As a result, the brain–skull displacement measurements are also of relatively coarse resolution and this may have impacted resulting boundary condition property assignment. Fifth, brain–skull displacements were matched uniformly around the periphery of the cerebrum in the sagittal plane, but coarse resolution data suggest there may be regional dependencies. Future studies may investigate more exact measurements of brain–skull displacement and regional differences in brain–skull displacement using higher resolution video, allowing for the determination finite element model sagittal plane boundary conditions by region. Finally, physical measurements of maximum brain–skull displacement and the corresponding finite element boundary condition were only optimized for peak angular velocities of ~52 rad/s in the sagittal plane. A peak angular velocity of 50 rad/s was chosen to match previous axial rotation transection studies in the 3- to 5-day-old piglet (Ibrahim et al., 2010) for directional comparison of brain–skull displacement. Furthermore, the second consecutive rotation was also conducted at a nominal peak angular velocity of 50 rad/s to simulate repeated events at similar loading magnitudes. Not surprisingly, our prior axial rotation transection studies reveal increases in peak angular velocity produce larger brain tissue strains (Ibrahim et al., 2010; Sullivan et al., 2015); and we may also expect larger brain–skull displacements at higher load levels. Whether the prescribed finite element model boundary condition between brain and skull also holds for other load levels is unknown. We recommend future physical sagittal plane transection studies at higher levels of peak angular velocity and acceleration to further inform biofidelic brain–skull displacement conditions.

As with any computational model, the finite element model of the porcine head utilized in this study presents several limitations. First, the brain was approximated as a homogeneous and isotropic material without differentiation between gray and white matter, although empirical studies show that the brain is indeed heterogeneous and anisotropic (Prange and Margulies, 2002; Ning et al., 2006; Chatelin et al., 2010). In addition, the brain shear modulus was assigned based on reverse engineering of brain tissue strains and validated using previous converged versions of this porcine finite element model, as measured in prior axial transection *in situ* experiments. Although the brain shear modulus employed in this model is similar to those measured *in vitro* and *in situ*, it is unknown whether determination of brain shear modulus based on tissue strains in sagittal plane transections would result in a dissimilar property measurement. However, the objective of these studies involved evaluation of relative motion between the brain and skull, and as such, accuracy of the strains within the brain tissue was of less importance. Nonetheless, it is unclear how modeling more detailed brain structures or matching brain shear modulus or tissue strains to those observed in sagittal plane transections may affect brain–skull displacement or determination of boundary condition properties (i.e., connector stiffness). Continued investigations into the mechanical properties of porcine brain may enhance the biofidelity of the boundary condition properties. Future studies may include modeling of the porcine brain with appropriate gray and white matter differentiation, and validation of brain tissue strain with measured physical transection brain strains. Second, as the model geometry was created by digitizing consecutive computed tomography images, there was some loss of geometric integrity near the cerebellum and brainstem. While brain–skull displacement was only measured around the periphery of the cortex in the physical transection specimens, it is unknown how the caudal geometric inaccuracies may affect the motion of the brain relative to the skull at the fore- and mid-brain in the finite element model. In addition, the brain gyri in this finite element model were eliminated to create idealized brain and inner skull surfaces (Coats et al., 2012). This smoothed brain surface is expected to have allowed greater brain–skull displacement than a model including gyri and sulci (Coats et al., 2012), but it is unknown how their presence may affect the determination of the general connector stiffness used to define the brain–skull boundary condition. Third, as described in Section “Construction and Simulation,” the finite element model was not scaled to the physical transection experiment subjects’ brain masses as this could not be recorded with the physical experimental setup. However, the specimens utilized in the physical transection experiments were from piglets of the same species, and similar age and weight as those used in development of the finite element model, providing a best approximation of brain mass given experimental restraints. Fourth, as described in Section “Construction and Simulation,” the number and distribution of parasagittal bridging veins included in this model were chosen based on data determined from human adult cadaveric specimens (Han et al., 2007), as these data are unavailable in the pig. It is unknown whether the number and distribution of parasagittal bridging veins is similar across species. Fifth, bridging veins were modeled as non-linear axial connector elements with longitudinal tension stress–stretch behavior because the primary loading modality of the bridging veins is thought to be longitudinal tension as the flaccid vessels are stretched between their attachment points at the brain and superior sagittal sinus. The resting state tension in the bridging veins is unknown. We assume that the bridging veins are taut, but not prestressed at the onset of rotation, while *in vivo*, there may be some axial pre–stress, a considerable amount of slack in the vessel between its brain and sinus attachment points, or the bridging veins may pull out of the soft brain tissue during an insult. It is also possible that the bridging veins may snag on or be entangled with other structures between the brain and skull, namely, the dura and arachnoid membranes and/or arachnoid trabeculae (Yamashima and Friede, 1984). In addition, shear loading may be present in the bridging veins at their brain and sinus attachment points. To date, no mechanical property testing has been performed on bridging vein tissue in shear. Future studies may investigate shear loading at the bridging vein attachment points to determine its role in bridging vein failure behavior. Sixth, a boundary condition defined by linear elastic connectors positioned between brain surface and inner skull surface nodes was employed based on prior findings that this boundary type provided an approximate combined response of the PAC and CSF such that brain tissue strain and brain–skull displacement were similar between physical and finite element estimations, and provided good capability of predicting intracranial hemorrhage. While the prior study compared several different representations of the brain–skull boundary condition, these have not been compared in the sagittal plane transection model, and it is unknown whether a different boundary condition may provide a better fit between physical and finite element model responses. Future models may also include separate representation of the PAC and CSF to simulate their material properties, and in the case of CSF, fluid properties such as incompressibility. These investigations, involving different model representations and responses of the CSF and PAC, are important to better define this boundary condition. In addition, no optimization algorithm was employed to find the chosen general connector stiffness. Thus, is it unknown whether the 46.133 N/m is a global optimum, although it provided the best fit of the different stiffnesses tested, did not substantially vary with bridging vein behavior, and provided an acceptable fit for both all brain-rigid dot pairs and superior-only brain-rigid dot pairs, resulting in a broadly acceptable general brain–skull connector stiffness for sagittal plane rotation, and as such, should only be used to model sagittal plane rotational insults. Investigators interested in modeling-specific conditions or regions may include algorithmic determinations of a global optimum general connector stiffnesses based on direction of head rotation, brain–skull displacement region of interest, and assumed bridging vein behavior. Finally, it is important to note that the optimized connector stiffness determined in this study is specific to the physical experimental data and finite element model employed. As such, it would need to be evaluated for use in other models or in other rotational directions. While imperfect, the value in this finite element modeling study lies in the use of experimental data for validation of brain–skull displacement in the pig. Investigators may use these data to understand possible outcomes in studies employing *in vivo* porcine models of TBI where use of live or cadaveric human studies present ethical roadblocks.

It is also important to note a few differences in analysis. In the axial physical transection experiments performed in 2008 and 2009, the baseline distance between brain and rigid dots was determined as the distance found in the first frame before motion, rather than the first 50 frames before motion in this series of sagittal physical transection experiments. We do not believe this contributed to our finding of smaller displacements in the sagittal rotations. In the axially transected finite element model, brain–skull displacement was matched to physical experiments using a linear regression with 95% confidence intervals for slope containing 1, and intercept containing 0, rather than assuming an intercept of 0 as was done in the sagittally transected studies. Similarly, we do not believe this contributed to our finding of lower finite element model general connector stiffness in sagittal rotations. Finally, in the axial transection studies, the boundary condition was validated separately in all three axial transection studies, rather than by pooled analysis, and it was not optimized, but rather estimated and found to be within acceptable criteria for linear regression similarity. Future studies may optimize the axial transection general connector stiffness and pool all studies together for improved statistical power, and this may result in a small change in the axial plane boundary condition stiffness, but it is still doubtful that any changes would be drastic.

### Conclusion

In summary, we have measured sagittal plane brain–skull displacements from an *in situ* model of the neonatal piglet head and determined a brain–skull boundary condition to match these displacements in a neonatal pig head finite element model. The data presented in this study provide critical insight into directional differences in rotational brain–skull displacement and will be employed in finite element models of the whole neonatal porcine and human infant heads simulating sagittal plane rotational insults to best predict tissue response, in particular the incidence of bridging vein failure.

## Ethics Statement

Animal care and euthanasia procedures were approved by the University of Pennsylvania Institutional Animal Care and Use Committee.

## Author Contributions

SP and SM contributed to conception and design of experiments; conduction of physical transection experiments; data interpretation; article revision and approval for publication submission; and agreement to be accountable for all aspects of the work, ensuring its accuracy and integrity. SP contributed to development of finite element model and conduction of finite element model simulations; data analysis; and draft of article.

## Conflict of Interest Statement

The authors declare that the research was conducted in the absence of any commercial or financial relationships that could be construed as a potential conflict of interest.
